# Ultrahigh energy storage in process-engineered NaNbO₃-based thin films with superior thermal and cyclic stability

**DOI:** 10.1038/s41598-025-05243-2

**Published:** 2025-07-01

**Authors:** Alexander M. Kobald, Herbert Kobald, Theresa Gindel, Ivana Panzic, Marco Deluca

**Affiliations:** 1 Materials Center Leoben Forschung GmbH, Vordernberger Straße 12, Leoben, 8700 Austria; 2 Silicon Austria Labs GmbH, Sandgasse 34, Graz, 8010 Austria

**Keywords:** Dielectric energy storage, NaNbO_3_, Thin film capacitor, Chemical solution deposition, Relaxor ferroelectric, Materials for devices, Materials for energy and catalysis

## Abstract

Dielectric thin film capacitors are essential for miniaturized electronics and energy storage systems, offering ultrafast charge-discharge rates and high reliability. However, achieving high energy density, efficiency, and stability in lead-free systems remains challenging, particularly with scalable and cost-effective methods. Here, we demonstrate relaxor sodium niobate-based thin films with Bi and Mg substitution, synthesized via optimized chemical solution deposition. By tailoring crystallization temperature and heating rate, we achieved a recoverable energy density of 37 J cm^−3^ and an efficiency of 80% at 2.45 MV cm^−1^. The films exhibit exceptional thermal stability, with energy density variation below 10% up to 310 °C, and superior charge-discharge stability beyond 16 million cycles at high fields. Microstructural engineering, involving grain size reduction and enhanced granularity, was critical for achieving a high Weibull breakdown strength (2.29 MV cm^−1^) and reliability. These films outperform previously reported sodium niobate systems, surpassing lead-based alternatives in environmental sustainability and scalability. Our approach highlights the potential of sodium niobate-based thin films for high-performance dielectric capacitors in harsh environments, offering a scalable pathway for environmentally sustainable energy storage technologies.

**Supplementary Information:**

The online version contains supplementary material available at 10.1038/s41598-025-05243-2.

## Introduction

Dielectrics are vital for modern electronic industry because of their capability to store and release charge, and therefore energy, under application and removal of an external electric field. Dielectric capacitors are increasingly valued for their high energy and power densities, combined with long operational lifetimes, making them ideal for applications such as hybrid electric vehicles, grid energy storage, medical defibrillators, and other high-power systems^1–6^. While electrostatic capacitors possess the highest power density, yielding much faster charge and discharge rates, they have low energy densities compared to electrochemical systems such as batteries and supercapacitors, lagging behind at least one order of magnitude^6,7^. Miniaturization and cost-cutting led to a rapid growth in research to enhance energy density, thermal and cyclic stability and reliability of dielectric capacitors to meet the demand of highly integrated and miniaturized electronic systems nowadays^2,4,6^. Non-linear dielectrics such as ferroelectrics, relaxors and antiferroelectrics are promising candidates to meet these requirements due to their large polarization response induced from the external electric field^3,8–12^. Chemical modification and phase, interface, microstructural and strain engineering have been successfully implemented to enhance their energy storage capability^6,12–19^. While substantial advancements have been made in significantly boosting recoverable energy density and efficiency, there has been less focus on studying temperature stability, charge-discharge cyclic stability, polarization fatigue, and overall reliability^15,18,20^.

**Fig. 1:**
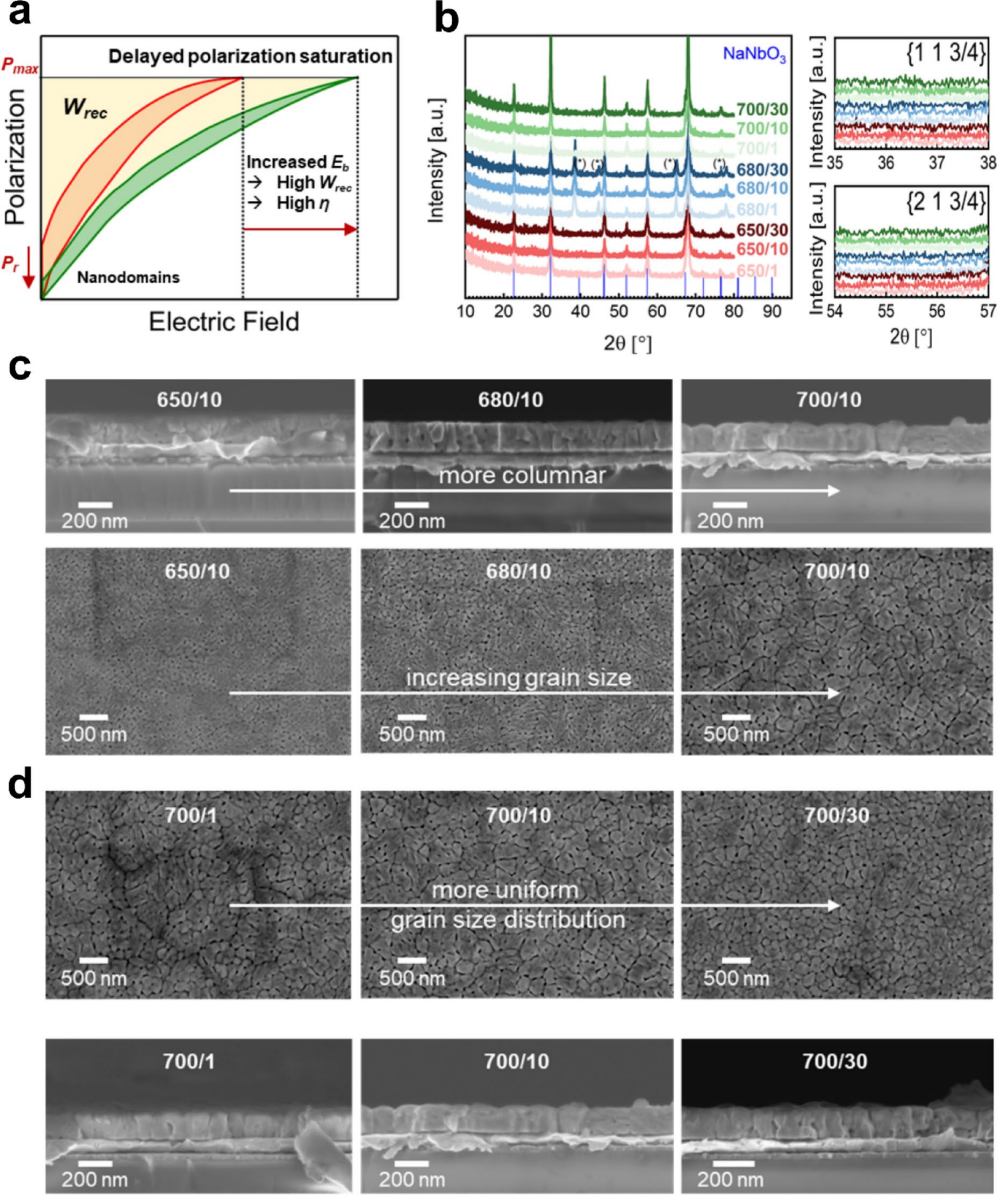
(**a**) Schematic representation of delayed polarization saturation. (**b**) GIXRD of all process variations of NN-BMN thin films with NaNbO_3_ reference (ICDD: 01-074-2456). No superlattice peaks at *{1 1 3/4}* and *{2 1 ¾}* are visible at 36.5 and 55.2°, respectively. Cross-section and surface SEM images for (**c**) different *T*_*cryst*_ at constant HR = 10 °Cs^− 1^ and (**d**) different HR at constant *T*_*cryst*_ of 700 °C.

The energy storage densities can be obtained from the integral region of the applied electric field (E) and the induced polarization (P) as:^21^

1 $$\:W{}_{rec}=\:{\int\:}_{{P}_{r}}^{{P}_{max}\:\:}EdP$$

2 $$\:W{}_{total}=\:{\int\:}_{0}^{{P}_{max}\:\:}EdP$$

3 $$\:W{}_{loss}=\:{W}_{total}-W{}_{rec}$$

4 $$\:\eta\:=\frac{W{}_{rec}}{{W}_{total}}\:x\:100\:\left[\%\right]$$

where *W*_*rec*_, *W*_*total*_ and *W*_*loss*_ are the recoverable, total and loss energy density and *P*_*max*_ and *P*_*r*_ are the maximum and remnant polarization, respectively, and η is the efficiency. The area inside the polarization – electric field (PE) hysteresis can be associated with the loss energy, dissipated mainly in heat. To achieve simultaneously high W_rec_ and η in a dielectric capacitor, the switchable polarization *ΔP = (P*_*max*_ *– P*_*r*_*)* and the electric breakdown strength E_b_ should be maximized while maintaining as little hysteresis as possible. By inducing chemical pressure from substitution, the hysteresis can be modified. One strategy is to induce relaxor-like behavior by breaking up the long-range ferroelectric ordering, introducing polar nanodomains. The nanodomains require less energy to be oriented in the external electric field, leading to a less pronounced hysteretic behavior and largely increased *η*, at cost of *P*_*max*_. This leads to the so-called delayed polarization saturation, which allows ultrahigh energy densities when E_b_ can be increased accordingly (Fig. 1a).

Ultrahigh energy density oxide thin films are typically produced using vacuum-based deposition techniques, which are costly, have limited scalability, and often involve low deposition rates. To ensure epitaxial film growth, single crystalline SrTiO_3_ (STO) substrates are often preferred over Si, allowing substantially increased polarization and therefore energy storage properties. However, the widespread adoption of this approach is hindered by the predominant use of Si-based technology in most electric and electronic systems. The necessity of vacuum for deposition can be circumvented by utilizing chemical solution deposition (CSD), which is highly cost-effective, scalable and chemically flexible^22^.

Most promising candidates from CSD for energy storage capacitors are based on toxic Pb^23,24^ with a research trend going towards Pb-free alternatives. Kwon et al.^25^ reported on BaTiO_3_ – Bi(Mg, Ti)O_3_ thin films from CSD deposited on Pt-Si wafers with *W*_*rec*_ = 37 J cm^− 3^ and good thermal stability up to 200 °C. Bi_0.5_Na_0.5_TiO_3_-based (BNT) systems show one of the highest energy densities among Pb-free CSD thin films, however, often at the cost of thermal and cyclic stability^13,26–28^. We recently showed a BNT-based material with ultrahigh energy density of 61 J cm^− 3^, 70% efficiency, thermal stability up to 180 °C and cyclic stability up to 10^5^ cycles from CSD^19^. For practical applications, the material must exhibit thermal and charge-discharge stability over a wide range. However, the use of BNT is often limited due to its restricted temperature stability up to 100–150 °C and significant polarization fatigue, where polarization diminishes with repeated cycles, leading to early degradation^13,19,29^. NaNbO_3_-based (NN) systems are seen as promising Pb-free candidates since NN has an antiferroelectric phase close to room temperature^30^. So far, only an electric-field-induced irreversible phase transition from antiferroelectric to ferroelectric was observed in pure bulk NN^31^, and reversible phase transitions at elevated temperatures in substituted systems^32,33^. No stable antiferroelectric NN thin films on Si substrates were reported because of the shallow energy difference between anti- and ferroelectric phases. However, chemical modification of NN to achieve relaxor states has been shown to be effective for energy storage in bulk ceramics^34,35^. Although NN is promising for energy storage^34^, its recoverable energy density and efficiency is comparably low because of low maximum polarization and high losses from leakage currents and hysteresis. The complexity of the phase structure of NN adds to the difficulty to find a range with minimal variation of properties for operation^30^. Especially the breakdown strength *E*_*b*_ is very low for thin films with values reported far below 1 MV cm^− 1^ accompanied by the cracking of the thin film because of the thermal mismatch^15^. Previous studies demonstrated the effectiveness of Mn substitution to reduce leakage currents in thin films because of its possibility to undergo reduction and oxidation easily, making low-concentration Mn-substitution one of the main strategies to reduce conductivity^36^. Partial Bi(Mg_2/3_Nb_1/3_)O_3_ substitution in NN bulk ceramics with Bi^3+^ and Mg^2+^ on the A- and B-site, respectively, was proven to enhance antiferroelectricity and to induce local random fields, introducing polar nanodomains, which lead to relaxor-like properties^37^ similar to Pb-based systems^38^.

In this work, we demonstrate 200 nm-thick 0.80NaNb_0.99_Mn_0.01_O_3_-0.20Bi(Mg_2/3_Nb_1/3_)_0.99_Mn_0.01_O_3_ thin films (NN-BMN) deposited on commercially-available Pt/TiO_2_/SiO_2_/Si substrates via chemical solution deposition (CSD). The thermal treatment was performed after every deposited layer and repeated for 5 times to achieve the final thickness with varying crystallization temperatures *T*_*cryst*_ of 650 to 700 °C and heating rates (HR) of 1–30 °Cs^− 1^ (Nomenclature: *T*_*cryst*_/HR) to investigate the processing sensitivity of this material system and get the best-performing thin film. A large increase of > 80% in breakdown strength was achieved by tailoring the heat treatment processing accordingly. Slim hysteresis and strongly increased breakdown strength were introduced by non-isovalent A- and B-site substitution and microstructural engineering, leading to a delayed polarization saturation, with an ultrahigh *W*_*rec*_ of 37 J cm^− 3^ and an efficiency of 80%. Additionally, the thin film capacitor exhibits high thermal stability (20–310 °C), high breakdown reliability (*E*_*b*_ = 2.29 MV cm^− 1^, *β* = 14.4), and excellent electrical fatigue properties (> 1.6 * 10^7^ cycles), making it suitable for applications at high temperatures and harsh environments.

## Experimental section

### Sample preparation

The 0.80NaNb_0.99_Mn_0.01_O_3_-0.20Bi(Mg_2/3_Nb_1/3_)_0.99_Mn_0.01_O_3_ were deposited on Pt/TiO_2_/SiO_2_/Si substrates (SINTEF, Norway) via chemical solution deposition (CSD). For synthesis of the precursor solutions, commercially available high-purity sodium(I) acetate (Na(CH₃COO), 99%, Carl Roth, Germany), bismuth(III) acetate (Bi(CH_3_COO)_3_, 99%, Alfa Aesar, USA), niobium(V) ethoxide (Nb(OCH_2_CH_3_)_5_, 99.95%, Sigma Aldrich, USA), manganese(II) acetate tetrahydrate (Mn(CH_3_COO)_2_ * 4 H_2_O, 99%, Sigma Aldrich, USA) and magnesium acetate tetrahydrate (Mg(CH_3_COO)_2_)*4 H_2_O, 98%, Carl Roth, Germany) were dissolved in acetic acid (C_2_H_4_O_2_, 100%, Carl Roth, Germany) and 2-methoxyethanol (C_3_H_8_O_2_, 99%, Carl Roth, Germany). Acetylacetone (C_5_H_8_O_2_, 99%, Sigma Aldrich, USA) and ammonia solution (NH_4_OH, 28–30%, Merck, Germany) were used as complexing agent and for pH adjustment, respectively. The A-site and B-site raw materials were dissolved in acetic acid and 2-methoxyethanol, respectively. The A-site solution was stirred at 80 °C for 2 h and the pH value was adjusted by adding ammonia until completely dissolved. The B-site solution was mixed at room temperature and stabilized with acetylacetone. Both A- and B-site solutions were mixed under stirring for 3 h. Finally, the concentration was adjusted to 0.3 mol L^−1^. Preparation of the stable precursor solution was performed in a dry N_2_ glovebox (Sylatech, Germany). The solution was aged 48 h before deposition on the Pt-substrates by spin-coating at 4000 rpm for 30 s (SPIN150i, APT GmbH, Germany). After coating, the thin film was dried 2 min at 200 °C, pyrolyzed at 450 °C for 5 min and crystallized at 650 °C, 680 °C and 700 °C with 1, 10 and 30 °Cs^− 1^ heating rate in a MILA50-5050 rapid thermal annealer (Advance Riko, Japan) in oxygen atmosphere. The process was repeated 5 times to achieve a total thickness of 200 nm.

### Characterization

The structural analysis was conducted using Grazing Incidence X-ray Diffraction (GI-XRD) with a Bruker D8 Advance (Karlsruhe, Germany), employing CuK_α_ radiation at 40 kV and 25 mA. The samples were examined in continuous mode across a 2θ angle range of 10° to 80°, with 0.01° increments, at a scan rate of 2° per minute and an incidence angle of 0.80°. The surface and cross-section morphology were investigated with a Zeiss AURIGA^®^-CrossBeam^®^ dual-beam SEM (Zeiss, Germany) and the energy dispersive spectroscopy was done with an X-Act detector (Oxford Instruments, UK). For point and area measurements, a measuring time of 10 s and an acceleration voltage of 5 kV were selected. In contrast, for element distribution images, a measuring time of 786 s and also an acceleration voltage of 5 kV were employed. Raman spectroscopy was performed with a WITec alpha300R spectrometer (WITec GmbH, Germany) with a grating of 1800 gr mm^−1^ and a 100x EC Epiplan-Neofluar DIC objective (Zeiss, Germany), using a solid-state laser with a wavelength of 532 nm and 10 mW applied power for the excitation. Atomic Force Microscopy (AFM) characterization was carried out using a CoreAFM system (Nanosurf, Switzerland) under ambient conditions. Non-contact (tapping) mode imaging was performed. Scan parameters were set with a nominal force of 15% and an acquisition time of 0.78 s over a 10 * 10 μm area, using a Tap300Al-G probe. The resulting images were processed with the Gwyddion software^39^. For electrical measurements, 250 μm radial Cr/Au electrodes were deposited through a shadow mask by electron-beam assisted thermal evaporation using a Korvus HEX Series TAU. The polarization vs. electric field (PE), impedance vs. frequency and cyclic stability were measured with an aixACCT TF3000 analyzer and a heating stage from 20 to 360 °C. The characteristic breakdown strength *E*_*b*_ was calculated by using a two-parameter Weibull distribution^40^.

## Results and discussion

In Fig. 1b, Grazing Incidence X-ray Diffraction (GIXRD) profiles of the NN-BMN films reveal the effects of varying processing conditions. For *T*_*cryst*_ of 650 °C and 700 °C, all peaks were assigned to the NaNbO_3_ pseudo-cubic perovskite phase (ICDD: 01-074-2456). Interestingly, *T*_*cryst*_ of 680 °C shows additional peaks at 44.6 and 64.9° 2θ for all HRs, which might be associated to a complex oxide formation with pyrochlore structure from Bi, Mg and Nb. This underlines the importance of process optimization in CSD in complex mixed oxide systems because of complicated thermodynamics. NaNbO_3_ thin films are known from literature to be able to exhibit a phase coexistence of ferro- and antiferroelectric phases in their pristine state under certain processing and temperature conditions^30,34^; however, in our substituted thin films, no direct evidence for such coexistence is observed. Due to the narrow energy gap between these phases, there is an irreversible phase transition from antiferroelectric to ferroelectric when the material is subjected to external electric fields^15^. Both Bi- and Mg substitution increase the experimental tolerance factor t and also decrease the difference in electronegativity^33^. This leads to a ferro-distortive behavior, stabilizing the ferroelectric phase over the antiferroelectric and influencing the phase fraction between the competing phases. The characteristic *{1 1 ¾}* and *{2 1 ¾}* superlattice reflections of the *Pbma* antiferroelectric P-phase at 36.5 and 55.2°, respectively, are not present in NN-BMN thin films (Fig. 1b), demonstrating the ferroelectric nature of these thin films and the absence of the antiferroelectric P-phase. The uniformly dispersed Bi and Mg ions are assumed to induce chemical disorder in the NaNbO_3_ system, likely resulting in breaking the long-range ferroelectric order into nano-sized domains with simultaneous high polarization because of the strong hybridization of Bi 6p and O 2p orbitals, as reported for similar bulk ceramics^35,37^. While direct domain observation is not provided here, the broad Raman peaks and slim hysteresis loops are consistent with such relaxor behavior. Therefore, this particular chemistry is highly interesting because of its relaxor properties in a NaNbO_3_ thin film. Raman spectroscopy confirms the highly disordered character of the system by revealing very broad peaks with no secondary phases present (Figure S1). Cross-sectional Scanning Electron Microscopy (SEM) images display the multilayer structure, consisting of the substrate with the bottom electrode and the 200 nm thick film deposited on top of it (Fig. 1c and d). Figure 1c shows both the cross-section and the surface morphology from SEM for a constant HR of 10 °Cs^− 1^ for all three *T*_*cryst*_. Cross-sectional analysis shows a change in growth mode corresponding to the crystallization temperature. Columnar grains connecting the top and bottom interface of the thin film capacitor are seen at 700 °C whereas decreasing the temperature leads to an increase in granularity. This disrupts the direct grain boundary pathways between the top and bottom electrodes, a desirable feature for reducing conduction-related losses along grain boundaries in practical applications. From the surface SEM, a crack-free surface with a uniform microstructure can be seen. An increase of grain size with *T*_*cryst*_ is evident, as expected from nucleation and growth kinetic theory. The grain size is reduced from around 400 nm^15^ to the 100 nm-range compared to pure NN thin films, since Bi^2+^ and Mg^2+^ act as nucleation centers, thereby increasing the nucleation rate during solidification. Energy dispersive Spectroscopy (EDS) was performed to confirm dispersion homogeneity of the solid solution on the nm-scale. Figure S2 shows that the substituents Bi, Mg and Mn are uniformly dispersed on the nm-scale inside the thin film with no visible aggregations at lower temperatures. A less uniform distribution is seen at 700 °C, which is assumed to be attributed to an increased elemental volatility at higher processing temperatures. The stoichiometry in at% with a 95% confidence interval was determined for all process conditions from the EDS data and compared to the theoretical stoichiometry of the composition and can be found in Figure S2. The A-site substituents (Na, Bi) fall within the margin of uncertainty of the theoretical stoichiometry. On the B-site, Nb is slightly over- and Mg under-stoichiometric, however, with a maximum deviation of 1 at% and 0.5 at% from the theoretical value, respectively. Good stoichiometry was achieved by counteracting the volatility of Na and Bi through the addition of excess ions during solution preparation with 10 mol% and 2 mol%, respectively. Oxygen is maximum 1 at% under-stoichiometric, which points to the presence of oxygen vacancies, and were counteracted with 1 mol% Mn substitution on the B-site, considering its good redox potential as discussed in the introduction. This allows good control over the microstructure of the CSD thin film without altering the stoichiometry or elemental distribution to an unwanted extend and allowing microstructural engineering to tailor properties of the final capacitor. Figure 1d demonstrates the effect of the heating rate at constant *T*_*cryst*_ on the surface and cross-section morphology. From the top view, a change in grain size uniformity was found with well-shaped grains around 100 nm at 30 °Cs^− 1^. Lower heating rates change the nucleation of the material and introduce differently sized grains, leading to a larger heterogeneity in grain size distribution, which is known to demonstrate worse breakdown strength and lower reliability.

**Fig. 2:**
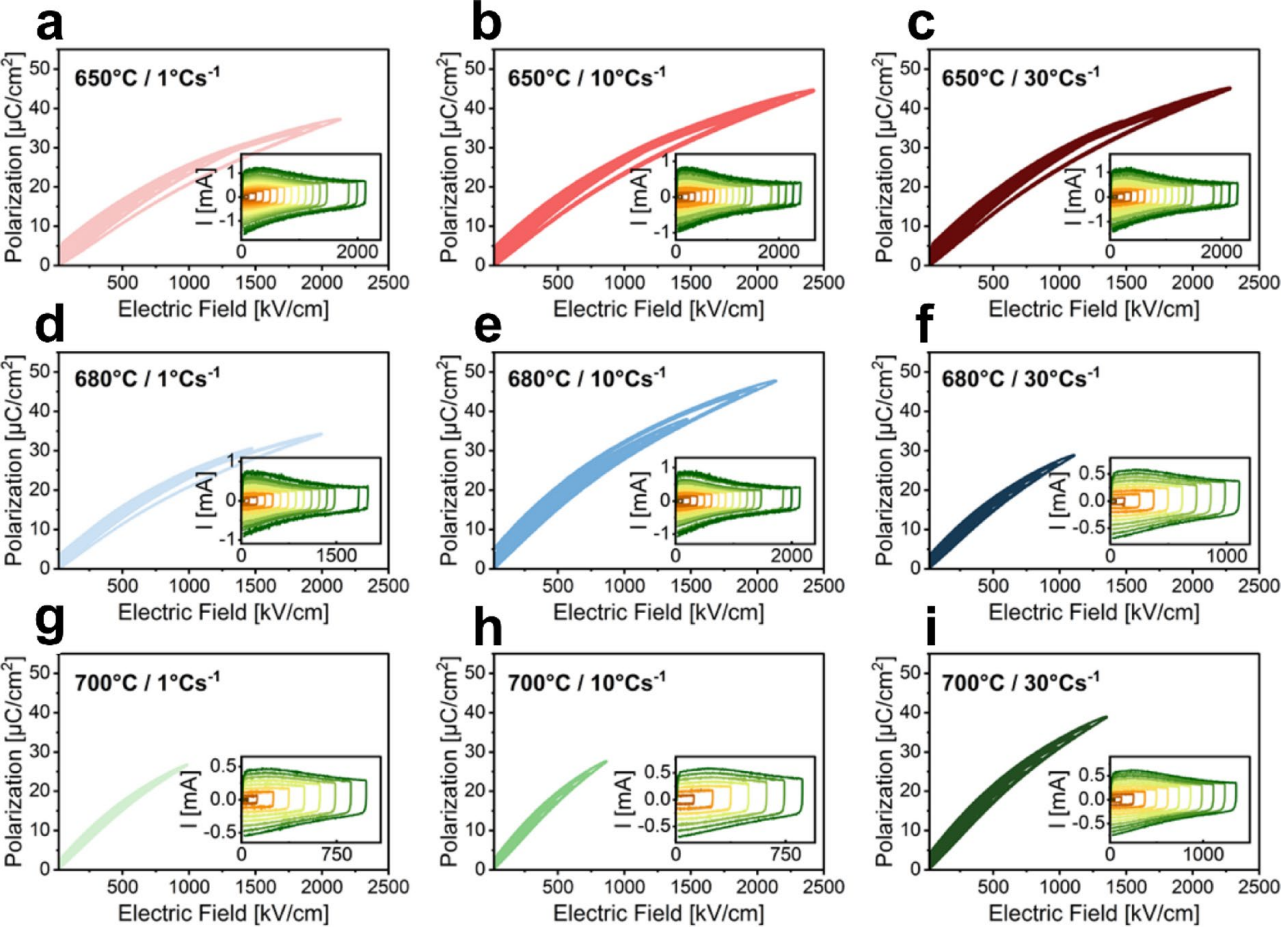
Unipolar PE hysteresis loops of NN-BMN thin films at (**a-i**) various *T*_*cryst*_ and HR combinations, measured at 10 kHz. The insets show the corresponding switching current (I-E) curves. The x-axis in the insets is the electric field and is identical to the main plots, thus omitted for clarity. The color gradient in the insets ranges from red to green, representing increasing maximum applied electric field values.

Figure 2a-i presents the unipolar PE hysteresis loops for the different combinations of *T*_*cryst*_ and HR (650/1, 650/10, 650/30, 680/1, 680/10, 680/30, 700/1, 700/10, 700/30) measured at 10 kHz with the corresponding switching current (I ~ dP/dt) signal inserted. This reveals the significant impact of small temperature changes and the heating rate. Hence, optimizing the thermal processing is essential for achieving high-quality thin films with superior energy storage properties, as CSD is a highly non-equilibrium nucleation and growth process. A strongly decreasing maximum applicable field before breakdown can be seen for increasing the crystallization temperature. Both *P*_*max*_ and *E*_*b*_ increase with higher HR for the high and low *T*_*cryst*_. At *T*_*cryst*_ of 650 °C, the variation of polarization with changing HR reduces and both medium and high HR yield superior properties. Interestingly, at medium *T*_*cryst*_, the medium HR of 10 °Cs^− 1^ stands out with highest *P*_*max*_ and *E*_*b*_.

**Fig. 3:**
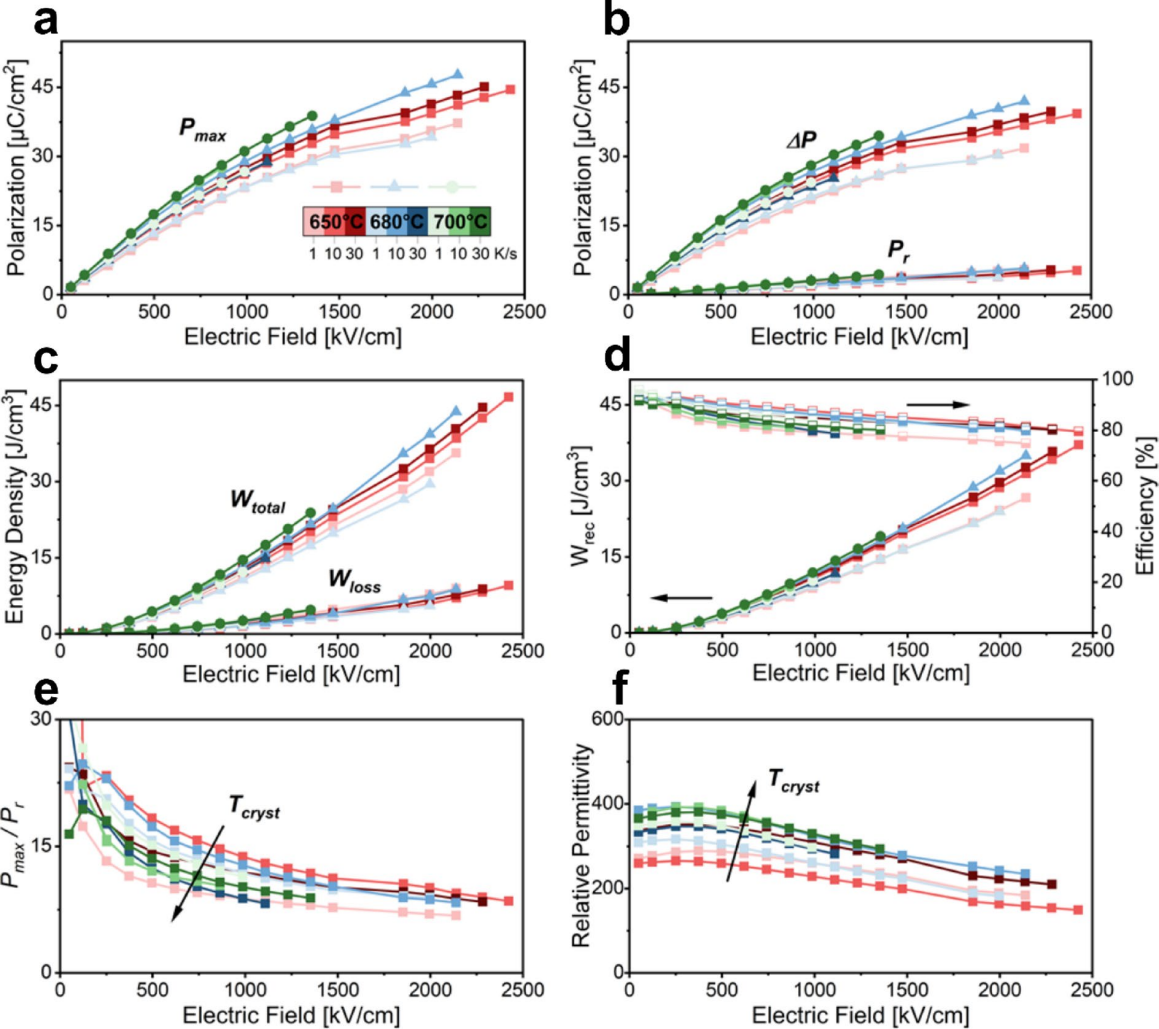
Electric field dependence of (**a**) *P*_*max*_, (**b**) *ΔP* and *P*_*r*_, (**c**) *W*_*total*_ and *W*_*loss*_, (**d**) *W*_*rec*_ and efficiency, (**e**) *P*_*max*_ */ P*_*r*_ and (**f**) relative permittivity for all process variation conditions measured at 10 kHz.

Figure 3a presents *P*_*max*_ as a function of the applied electric field. A clear trend of higher *P*_*max*_ with higher *T*_*cryst*_ is noticeable, coming presumably from a higher degree of crystallinity as expected at higher temperatures, with 39 µC cm^−2^ for 700/30 and from the more columnar grain structure, which is known to show higher polarization values. It is also noteworthy that *P*_*max*_ is distinctly lower for every tested temperature at the low heating rate of 1 °Cs^−1^ at equivalent fields with values down to ~ 30 µC cm^−2^ at 1.35 MV cm^−1^. This underscores the necessity of rapid thermal annealing for CSD-produced films to achieve necessary HR > 1 °Cs^−1^. The low *T*_*cryst*_ of 650 °C shows in general for all HR the best trade-off between high *P*_*max*_ > 40 µC cm^−2^ and high *E*_*b*_ > 2 MV cm^−1^. Since the *W*_*rec*_ of non-linear dielectrics is dependent on the integral interval between *P*_*r*_ and *P*_*max*_, the switchable polarization *ΔP* and *P*_*r*_ are indicators of high energy storage densities. Figure 3b shows both *ΔP* and *P*_*r*_ over the electric field. The same trend as for *P*_*max*_ is found for *ΔP*, even though *P*_*r*_ is increasing for increasing *T*_*cryst*_. In general, the material composition demonstrates low *P*_*r*_ < 4 µC cm^−2^ for all tested conditions. In Fig. 3c-d, the calculated *W*_*total*_, *W*_*loss*_, *W*_*rec*_ and *η* over the applied electric field for all process conditions can be found. The best film at 700 °C with high HR of 30 °Cs^−1^ (700/30) has a high *W*_*rec*_ of 19 J cm^−3^ at 1.35 MV cm^−1^, setting the limit for the high crystallization temperature films. Since the rated voltage for applications is always much lower than *E*_*b*_ to ensure operation without critical failure, a high enough *E*_*b*_ is important. The low *T*_*cryst*_ films show at all tested heating rates *E*_*b*_ > 2 MV cm^−1^, demonstrating little sensitivity to the heating rate and therefore higher processing reliability. This high *E*_*b*_ allows a large operation window up to high electric fields without approaching the *E*_*b*_ closely. The energy storage properties of 650/10 sample are superior because of the high *E*_*b*_, reaching a high *W*_*rec*_ of 37 J cm^−3^ at a field of 2.45 MV cm^−1^ with a simultaneously high *η* of 80%. This large increase in *W*_*rec*_ of 95% compared to the best film at 700 °C (700/30) can be explained due to the largely delayed polarization saturation commonly found in relaxors, shifting *P*_*max*_ to higher fields and therefore increasing energy storage at maximum field, however slightly lowering the properties at lower fields compared to the films crystallized at higher temperatures. The capability of reaching this high *E*_*b*_ can be attributed to the combination of both decreased grain size and reduced conduction pathways connecting top and bottom electrode by introducing a higher degree of granularity along the cross-section, leading to a higher breakdown strength. Figure 3e shows the ratio *P*_*max*_ */ P*_*r*_, which is another very strong indicator of energy storage performance and can be taken to visualize the delayed polarization saturation. The ratio starts to flatten and reach its high saturation ratio of ~ 9 at much higher fields for lower *T*_*cryst*_, e.g. at > 2 MV cm^−1^ for 650/10 compared to 1.3 MV cm^−1^ for 700/30. The change of relative permittivity over the operating electric field is presented in Fig. 3f. For dielectric energy storage applications, the tunability, represented by the change of permittivity over electric field, should be as low as possible to increase the operation window with approximately constant material properties, which is at maximum 40% at an *E*_*b*_ of 2.4 MV cm^−1^ for 650/10. A positive correlation between *T*_*cryst*_ and relative permittivity is noticeable, as both decrease together when the processing temperature is reduced. The delayed polarization saturation, stable permittivity with minimal tunability, and slim hysteresis (low remnant polarization) yield high recoverable energy density and efficiency, making the 650/10 film highly promising for applications.

**Fig. 4:**
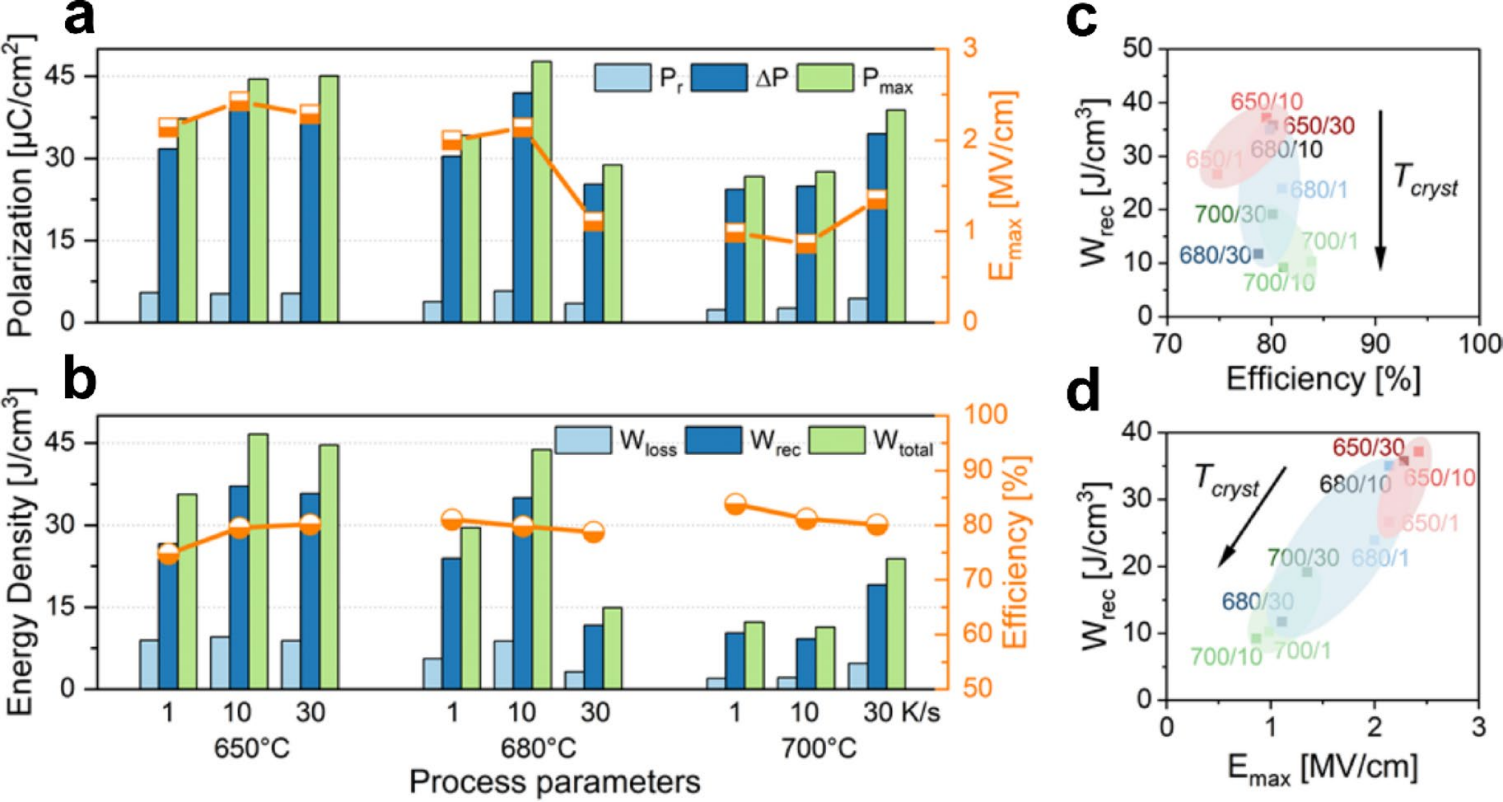
Process-Property relations: (**a**) Polarization properties and maximum applied field *E*_*max*_ and (**b**) energy storage properties and efficiency for all tested processing parameter combinations. Key performance comparison of all tested thin films regarding (**c**) *W*_*rec*_ vs. *Efficiency* and (**d**) *W*_*rec*_ vs. *E*_*max*_.

Figure 4 presents the discussed findings summarized for all process parameter variations performed at their corresponding maximum applied field. A clear decreasing trend in energy storage (*W*_*rec*_) and polarization (*P*_*max*_) is observed with increasing *T*_*cryst*_. It is noteworthy that the efficiency is close to 80% for all samples, however, the lower *T*_*cryst*_ films operate at much higher fields and still reach the same efficiency. The key performance indicator graphs *W*_*rec*_ vs. *η* and *W*_*rec*_ vs. *E*_*max*_ are shown in Fig. 4c and d, respectively. They clearly indicate that this material exhibits approximately (80 ± 10)% efficiency at the maximum field, regardless of process optimization; however, the *E*_*b*_ varies significantly. *W*_*rec*_ is significantly influenced by process conditions, primarily driven by the nearly linear increase in the maximum applicable field with decreasing temperature and the resulting delayed polarization saturation.

**Fig. 5:**
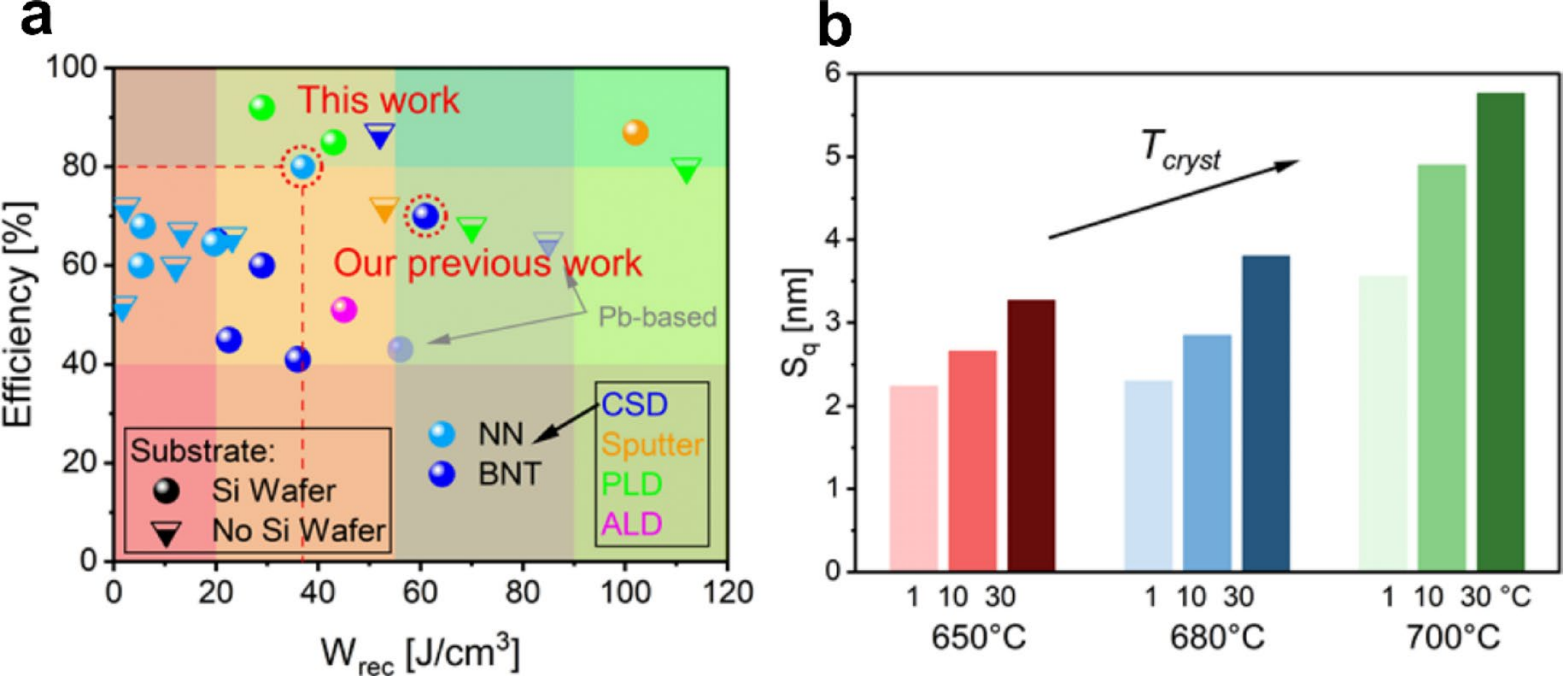
(**a**) Comparison of dielectric energy storage capacitors regarding used substrate and technique^4,13,15,19,23,24,26–28,41–55^. (**b**) Mean-root-square roughness S_q_ from AFM measurements for tested process parameters.

The highest energy storage values reported so far for NN-based thin films wafers are *W*_*rec*_ = 19.64 J cm^− 3^ with *η* = 64.5% on Si^41^ and *W*_*rec*_ = 23.1 J cm^− 3^ and *η* = 66% on STO^42^. Hence, our thin films outperform the highest NN-based thin films with 88% and 60% higher *W*_*rec*_, respectively, and a higher η of 80%, even surpassing most BNT-based thin films from CSD regarding both *W*_*rec*_ and especially *η* (cf. Figure 5a). These results demonstrate that our films achieve the highest recoverable energy density and efficiency among CSD-derived NaNbO_3_-based thin films.

For applications, a smooth surface is highly favored because of the superior adhesion and interface towards the top electrode. Future developments could include thin film ceramic capacitor devices inspired by the concept of a multilayer ceramic capacitor^10^. Hence, the interface roughness of the dielectric layers with its top electrode builds up the foundation for the subsequent layer of the multilayer structure, making roughness in the very low nm-range essential. Figure 5b shows the mean-root-square roughness S_q_ derived from the AFM measurements (Figure S3, Table S1) as a means to investigate the compatibility with applications. As seen, a positive correlation between increasing temperature and heating rate with increasing roughness is noticeable. This underlines the outstanding properties of the 650/10 sample, combining high energy storage properties with high breakdown strength and ultralow roughness of ~ 2.5 nm, which is a half compared to the best energy storage sample at 700 °C (700/30).

**Fig. 6:**
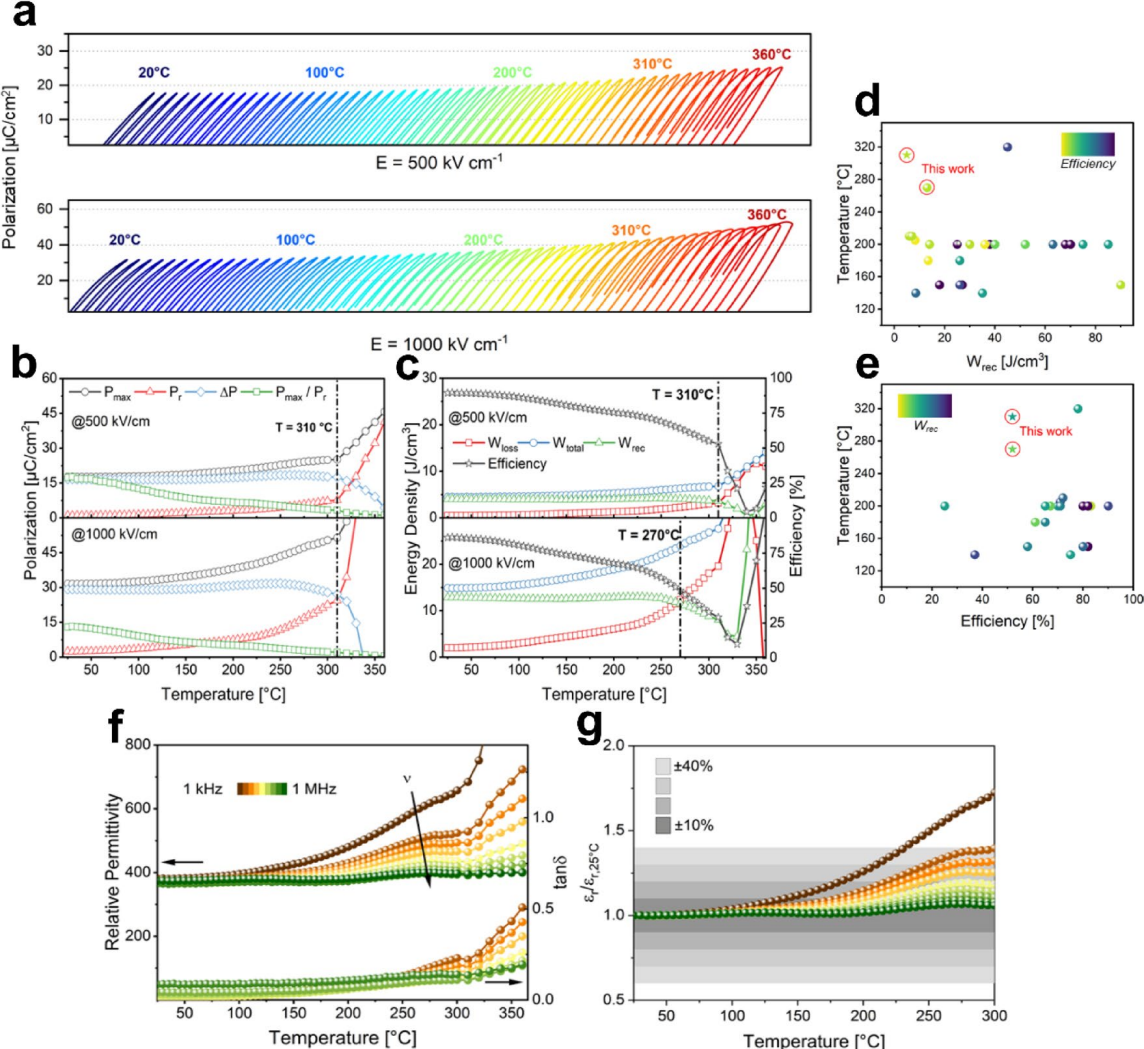
Temperature-dependent PE loops of BMN-NN thin films from 25 to 360 °C at (**a**) 500 and 1000 kV cm^− 1^ with 5 °C temperature steps (blue to red). Temperature stability of (**b**) polarization and (**c**) energy storage properties. Literature comparison of thin film dielectrics for energy storage at reported highest temperatures for (**d**) *W*_*rec*_ and (**e**) *efficiency*. (**f**) Relative permittivity *ε*_*r*_ and loss tangent *tanδ* and (**g**) relative change of *ε*_*r*_ to room temperature value at 25 °C *ε*_*r,25 °C*_.

Thermal stability is also an important factor for the application of dielectric energy storage capacitors. The temperature stability of the best thin film, NN-BMN 650/30, was studied by measuring unipolar PE hysteresis at low (500 kV cm^− 1^) and high (1000 kV cm^− 1^) fields and can be seen in Fig. 6a. The unipolar P-E loops stay constant up to 150 and 180 °C for low and high fields, respectively with no changes visible. After this point, the maximum polarization slightly increases together with the remnant polarization and stays within negligible variations up to 270 and 310 °C, respectively. Afterwards, the remnant polarization increases rapidly, marking the increase of temperature-induced leakage current. Polarization and calculated energy storage densities for 650/10 can be seen in Fig. 6b and c, respectively. No abrupt changes in polarization are visible up to a temperature of 310 °C, making this thin film highly temperature stable. At high fields a slight but steady increase in both *P*_*max*_ and *P*_*r*_ is noticeable, however, leading to a nearly constant *ΔP*. At *T * > 310 °C, polarization increases sharply at both tested fields, signaling a diffuse phase transition. At low fields, *W*_*total*_ and *W*_*loss*_ are constant up to 220 °C before slightly increasing. *W*_*rec*_ stays within < 10% variation up to 310 °C at low and up to 270 °C at high fields, making this thin film highly suitable for high temperature applications at fields around 1 MV cm^− 1^, yielding a *W*_*rec*_ = 13 J cm^− 3^ with 52–86% efficiency and surpassing the highest reported NN-based temperature stability of 210 °C by far^42^. Fig. 6d and e present a comparison of recoverable energy densities and efficiencies of dielectric energy storage thin films reported in literature at elevated temperatures^4,11,18,42,52,56–75^. These plots highlight that while several materials maintain higher energy densities, our NN-BMN thin film system combines outstanding temperature stability with competitive performance, setting it apart from other state-of-the-art Pb-free systems.

In Fig. 6f, the relative permittivity is plotted over the measured temperature range. It shows the typical frequency dispersion of a relaxor in the proximity of T_m_ (250–275 °C) together with diffuse phase transitions, as also shown for a bulk NN-BMN system^37^. Relative permittivity varies by less than 40% up to 300 °C across frequencies from 10 kHz to 1 MHz with dielectric loss *tanδ* < 0.013 at 10 kHz. (cf. Figure 6g).

The simultaneous increase in dielectric permittivity and loss tangent with temperature, as seen in Fig. 6c, may be partly attributed to Maxwell–Wagner polarization mechanisms^76^. This interfacial polarization effect arises due to the build-up of space charge carriers at grain boundaries, particularly in heterogeneous or granular systems, where grains and grain boundaries possess different electrical conductivities. Such behavior is commonly modeled using effective medium approximations (EMAs), which treat the dielectric film as a composite of semiconducting and insulating phases^77–79^. At elevated temperatures, thermally activated charge carriers are more mobile and accumulate at interfaces, leading to enhanced permittivity and dielectric losses. This granular-type polarization effect becomes prominent in thin films with microstructural inhomogeneities, such as those induced by non-isovalent substitutions or process-induced grain boundary variations, which is supported by the observation of a high degree of granularity in the SEM investigation (cf. Figure 1c and d). Figure S2 demonstrates good chemical homogeneity for the thin films on the nm-range with some degree of Mg segregation, which gets more prominent at higher processing temperatures (and therefore higher degree of granularity). A more extensive investigation of ferroelectric nanoparticles in a polymer matrix using different EMA models was done by Pylypchuk et al.^80^.

Moreover, the reduced permittivity compared to bulk relaxor ceramics can be explained by a combination of the size effect^81^ and microstructural features. Thin films often exhibit suppressed dielectric response due to the reduced number of polar nanoregions and limited domain wall mobility imposed by mechanical and substrate-induced clamping as well as areduced atomic polarizability with decreased dielectric thickness. Additionally, Mg²⁺ ions may tend to segregate toward the grain boundaries during crystallization, forming a core–shell type structure. These grain boundary shells typically have lower permittivity and higher resistivity, which reduces the effective dielectric response while simultaneously enhancing the breakdown strength^82^. The resistive shell regions act as barriers to conduction, mitigating local field intensification and enhancing dielectric reliability. This microstructural design, in combination with the inherent size effect in thin films, provides a compelling explanation for the moderate dielectric permittivity and the excellent breakdown performance observed in our system.

As shown in Figure S4, the relative permittivity is also very constant over a large frequency range at room temperature (< 5% variation). The remarkable temperature stability of the energy and dielectric properties is ascribed to the high phase transition temperatures of NN. However, pure NN shows seven phases, comprising the aristotype and six hettotypes^30^, making this system extremely complex and hard to handle for applications. By introducing Bi- and Mg-substitution and using thin film technology, the phase transitions get very diffuse and only high temperature phase transitions (*T* > 300 °C) are detected, therefore making this substituted system highly suitable for operation in harsh conditions at high temperatures and large frequency ranges.

**Fig. 7:**
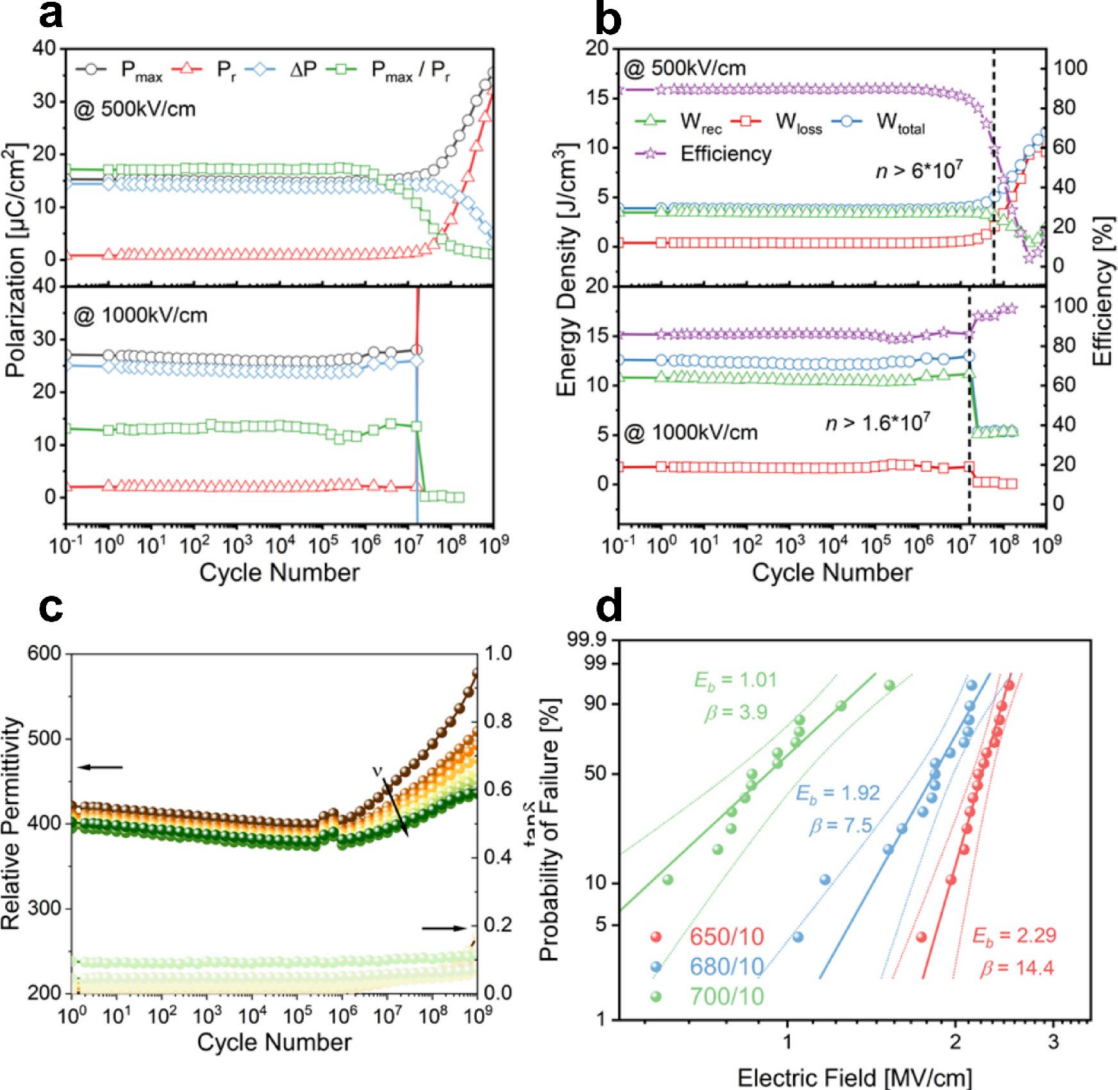
Fatigue behavior of (**a**) Polarization, (**b**) Energy Density and Efficiency and relative permittivity (**c**) as a function of cycle number at 500 or 1000 kV cm^− 1^ and measured at 10 kHz. (**d**) Two-parameter Weibull distribution analysis of breakdown strengths *E*_*b*_ for 650/10, 680/10 and 700/10 thin films.

Similar to the temperature stability, the 650/10 thin film demonstrates high cyclic stability at low and high fields of 500 and 1000 kV cm^− 1^. The fatigue behavior of the polarization and energy density were tested by applying a 10 kHz triangular waveform for 10^x^ (x = 1, 2, 3, …, 9) cycles with 5x measurements at the same field and frequency each decade (cf. Figure 7a, b). *W*_*rec*_ and *η* stayed constant up to > 6*10^7^ cycles at low fields and > 1.6*10^7^ cycles at high fields. Degradation began at higher cycle numbers, culminating in dielectric breakdown beyond 10⁸ cycles. This demonstrates superior charge-discharge stability, exceeding the 5 × 10⁶ cycles reported for a comparable NN-based system^15^. From Fig. 7c, one can see the change of relative permittivity over the cycle number. The relative permittivity slightly decreases up to 10^6^ cycles and afterwards starts to show changes related to the observed degradation. Interestingly, the dielectric loss stays low and just increases for the lower measured frequencies at high number of cycles. To test the reliability of the breakdown strength, a two-parameter Weibull analysis was performed with 15 capacitors to failure and fitted to a standard Weibull distribution:^40^

5 $$\:{X}_{i}=\text{l}\text{n}\left({E}_{i}\right)$$

6 $$\:{Y}_{i}=\text{ln}\left(-{ln}\left(i-\frac{1}{n+1}\right)\right)$$

where i denotes the rank and n the total number of data points. The result can be found in Fig. 7d. A statistical value for *E*_*b*_ and the Weibull modulus *β*, which represents the dispersion in the data were obtained from the fitting. For comparison, the best performing thin film 650/10 as well as 680/10 and 700/10 were analyzed to statistically evaluate the influence of *T*_*cryst*_ on *E*_*b*_. We found the characteristic *E*_*b*_ to be 1.01 MV cm^− 1^ (*β* = 3.9), 1.92 MV cm^− 1^ (*β* = 7.5) and 2.29 MV cm^− 1^ (*β* = 14.4) for 700/10, 680/10 and 650/10, respectively. An increase of *E*_*b*_ of 126% is observed from 700/10 to 650/10 together with a large increase in Weibull modulus *β*, which signals lower dispersion of the breakdown strength and subsequently higher reliability of *E*_*b*_.

## Conclusion

In this work, we investigated the effects of crystallization temperature and heating rate on the microstructure and properties of relaxor sodium niobate thin films, co-substituted with Bi and Mg. Optimizing these processing parameters disrupted vertical columnar grain structures, reduced surface roughness, improved grain size uniformity, and maintained stoichiometry. This allowed delayed polarization saturation, leading to a recoverable energy density of 37 J cm^−^³ with 80% efficiency at 2.45 MV cm^− 1^ and a breakdown strength of 2.29 MV cm^− 1^. These films exhibit exceptional thermal stability, with recoverable energy density varying less than 10% up to 310 °C, and remarkable charge-discharge stability, maintaining performance beyond 16 million cycles at high fields. Compared to previously reported sodium niobate-based systems, these thin films represent a significant advancement in energy storage performance, setting new benchmarks for both recoverable energy density and reliability. Our findings highlight the potential of sodium niobate-based thin films for use in high-temperature and harsh-environment applications, such as automotive power systems, grid storage, and aerospace electronics. Future research could focus on scaling this approach to multilayer architectures, extending thermal stability further, and exploring additional substitutions to fine-tune dielectric and energy storage properties.

## Electronic supplementary material

Below is the link to the electronic supplementary material.

Supplementary Material 1

## Data Availability

All data supporting this study and its findings are available within the article and its supplementary information. The raw data that supports the study within this article and other findings related to this study are available from the corresponding author upon reasonable request.
