# Supplementary material for: Ultrahigh energy storage in process-engineered NaNbO₃-based thin films with superior thermal and cyclic stability
Source: Sci Rep. 2025 Jul 1;15:21617. doi: 10.1038/s41598-025-05243-2 (PMC12216296; doi:10.1038/s41598-025-05243-2)
Supplement: Supplementary file 1 — Supplementary Material 1 [file 41598_2025_5243_MOESM1_ESM.docx]

Supporting Information

**Ultrahigh Energy Storage in Process-Engineered NaNbO₃-Based Thin Films with Superior Thermal and Cyclic Stability**

Alexander M. Kobald^a*^, Herbert Kobald^a^, Theresa Gindel^a^, Ivana Panzic^a^, Marco Deluca^b^*

^a^Materials Center Leoben Forschung GmbH, Roseggerstraße 12, 8700 Leoben, Austria

^b^Silicon Austria Labs GmbH, Sandgasse 34, 8010 Graz, Austria

Raman spectroscopy confirms strong peak-broadening, which can be associated to an increased disorder in the system, typical for relaxor compositions and complex oxides with the typical NaNbO_3_-related modes visible. [1]


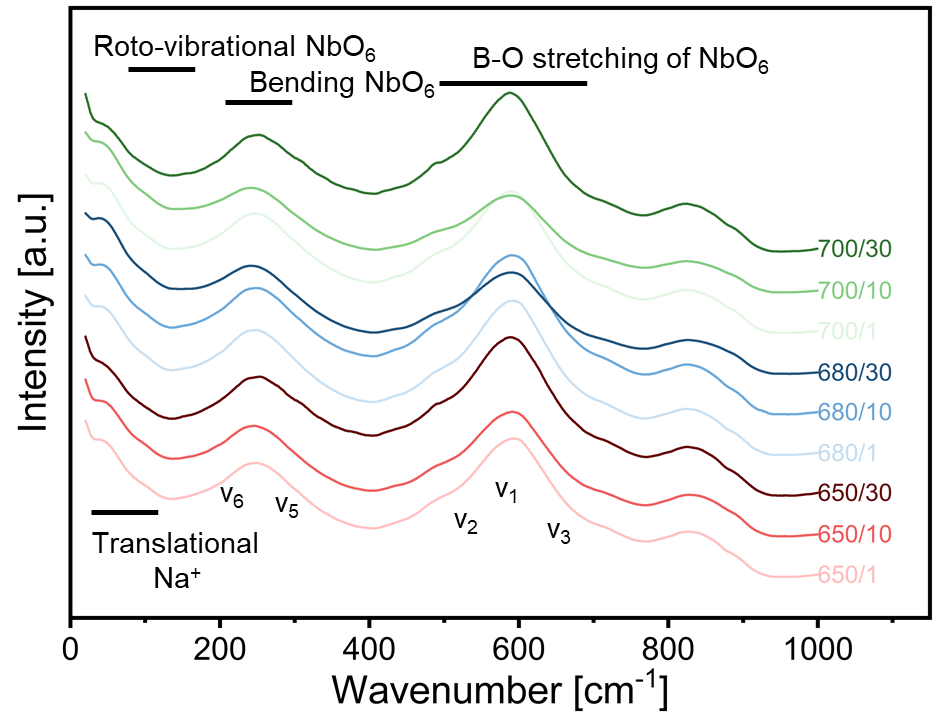


**Figure S1.** Raman spectra of NN-BMN thin films across varied crystallization temperatures from 650 °C to 700 °C and varied heating rates from 1 to 30 °Cs^‑1^ recorded at room temperature.


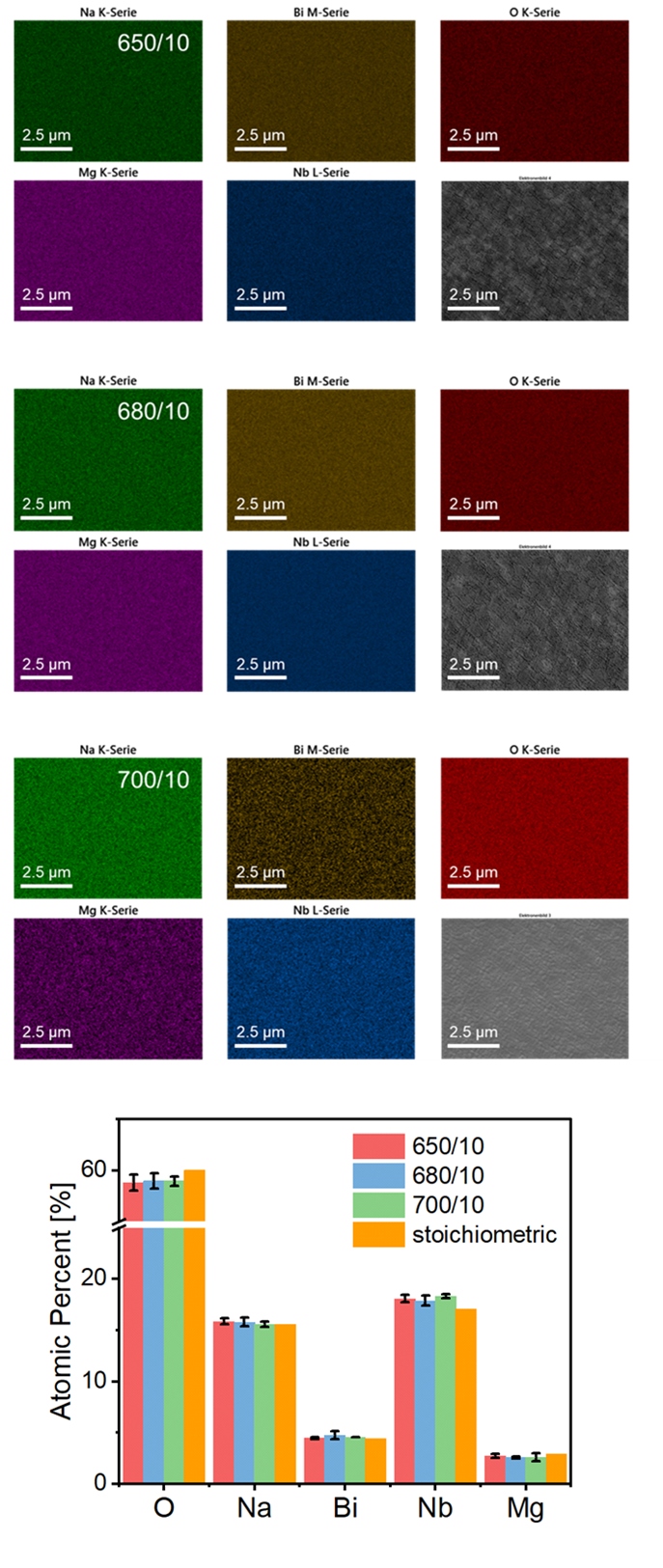


**Figure S2.** EDS Spectra with elemental distribution maps for 650/10, 680/10 and 700/10 thin films at 10 °Cs^‑1^ heating rate and comparison of stoichiometry at different crystallization temperatures.

**Table S1.** Mean-root roughness *S_q_* values from the AFM measurements.

| Sample | S_q_  [nm] |
| --- | --- |
| 650/1 | 2.238 |
| 650/10 | 2.662 |
| 650/30 | 3.269 |
| 680/1 | 2.299 |
| 680/10 | 2.843 |
| 680/30 | 3.803 |
| 700/1 | 3.553 |
| 700/10 | 4.895 |
| 700/30 | 5.762 |


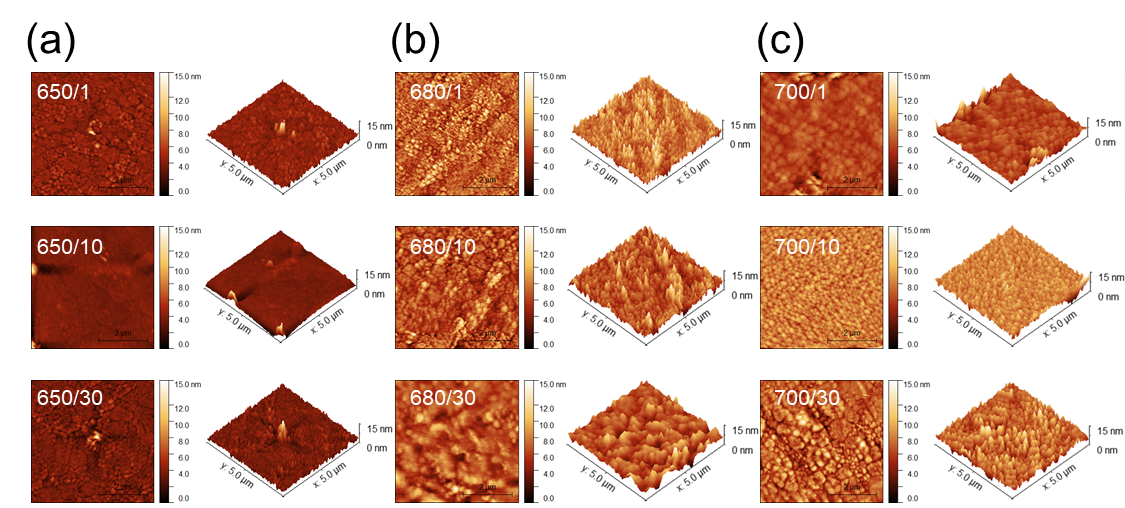


**Figure S3.** 2D and 3D atomic surface morphology images across varied *T_cryst_* (a)-(c) and varied heating rates from 1 to 30 °Cs^‑1^.


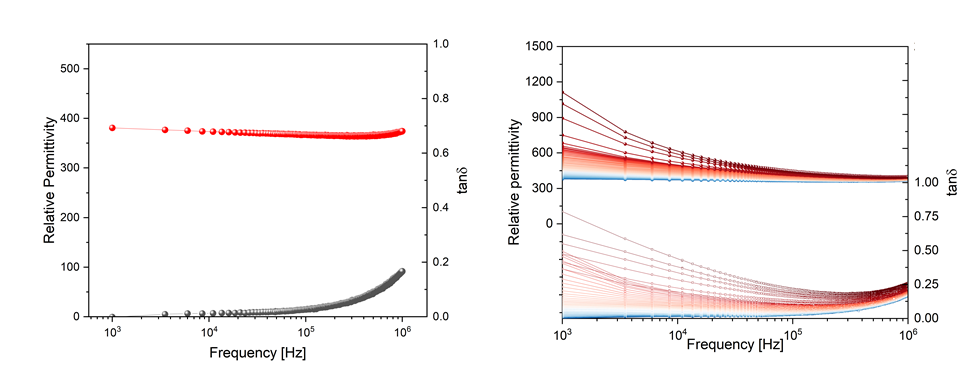


**Figure S4.** (a) Relative permittivity and dielectric loss *tanδ* vs. frequency at room temperature. and (b) development over temperature from 20 to 360 °C.
